# Supplementary material for: Spatial heterogeneity ensures long-term stability in vegetation and Fritillaria meleagris flowering in Uppsala Kungsäng, a semi-natural meadow
Source: PLoS One. 2023 Mar 8;18(3):e0282116. doi: 10.1371/journal.pone.0282116 (PMC10045606; doi:10.1371/journal.pone.0282116)
Supplement: S1 Appendix — (PDF) [file pone.0282116.s001.pdf]

Appendix S1. Hydrology and management at Uppsala Kungsäng.

Kungsängen Nature reserve is situated about 3 km south of Uppsala along the eastern shore of the River Fyrisån. The size of the reserve, partly a Natura 2000 site, is 12.5 ha, and it is situated between 1.07 and 2.57 m a.s.l. recalculated from Zhang (1983) to the present height system RH2000.

Kungsängen was in the 18th century situated closer to the sea level than at present, as the area is subjected to upheaval (currently about 6 mm yr^{-1}). Several seashore plant species were at that time still growing here (Sernander, 1948, pp 62–65). Land upheaval is slightly higher, ca 0.5 mm yr^{-1}, (Lantmäteriet Landhöjningsmodellen NKG2016LU) at the meadow than at the outflow in Stockholm to the Baltic Sea. On Rudbeck's map from the second half of the 17th century the Kungsängen area is marked as a wet or moist area (Sernander, 1948, Fig. 1). On a map from the 1730s (Yckenberg & Lindberg, 1732–1735, reproduced in Sandberg, 1948, Fig. 42) the lowest part of the present-day nature reserve was a wet meadow, where the text on the map includes plant names as "starr" (*Carex*), and "fräken" (*Equisetum*). The water level of the meadow is influenced by the water level of the river, which at high water is influenced by the bay Ekoln, part of the large Lake Mälaren, 6 km downstream (Zhang 1983, p. 24), by water from the surroundings and by precipitation. Before the first water regulation of Lake Mälaren in Stockholm, which began in 1940 and was completed in 1968 (Stockholms hamnar, 2021), the water level in Mälaren varied between the extreme values 0.13 (in 1939) and 2.26 (in 1853) m a.s.l. (System RH2000) (SMHI, 2012). In 1964, a new regulation plan of Lake Mälaren was decided and from 1966 the water level should vary between 0.69 m and 1.39 m a.s.l. (System RH2000). However, these efforts have partly failed, as between 1968 and 2019 the minimum level was 0.41 m and the maximum level 1.42 m, and the mean water level 0.86 m (System RH2000) (Mälarens vattenvårdsförbund, 2014; Stora_sjöar_statistik_2019).

Lake Mälaren's high water level, measured in Stockholm, is higher than the lowest parts of the meadow, and the meadow can be inundated during autumn, winter and spring (cf. Zhang, 1983, p. 25). Photos from April 1942 and April 1982 (Sandberg, 1948, p. 153, Fig. 34; Zhang, 1983, p. 24, Fig. 16) show that parts of the meadow were inundated. The altitude of the vegetation plots in Profile 1 was 0.62 and 1.92 m a.s.l, system 1900 (Sandberg, 1948), corresponding to 1.24 m and 2.54 m a.s.l. in system RH2000.

The water level in the River Fyrisån measured between 1938 and 1980, at a station (no longer in use) situated between Kungsängen and the bay Ekoln, showed the highest levels during April and the lowest during July–October and again higher towards winter with a new, but lower peak in December (Zhang, 1983, Fig. 15; cf. Hallgren & Sandsborg, 1968; Zhang & Hytteborn, 1985, Fig. 2). Also, the bay Ekoln has a narrow connection to the main part of Lake Mälaren and therefore its high water level may be higher than the level measured in Stockholm. The lowermost part of the reserve was regularly inundated, i.e., parts of Profiles 1 and 4, whereas the highest parts of the meadow have never been inundated during the period we are discussing.

Overview of the management of Uppsala Kungsäng.

Source	Time	Physical activity	Period for		Notes
			Mowing	Grazing	
Hebbe 1936	AD 700	Raised above sea level			
Hebbe 1936	From 1609		August	September	
Rudbeck 1679 in Hebbe 1936	1679		13 August	After mowing	Uppsala people were allowed to keep their cattle on the meadow after mowing; ditches were completed, funnelling water from the arable fields east of Kungsängen. These ditches may have lowered the water level of the meadow
Hebbe 1936	1700 century		Late July	After mowing	In wet years mowing was later
Sernander 1948	1942		Annual mowing?	Grazing the whole summer	
Zhang 1983	1979–83		ca 24 June	After mowing and onwards	As the production was higher early in the season on Kungsängen than on the surrounding pastures, the farmer wanted to start grazing as early as possible
M. Aronsson, field notes	1994–95		Late August	After mowing	
S.-O. Borgegård, unpubl.	1993	Cutting of tussocks in the wettest parts			
Länsstyrelsen i Uppsala län, 1999	1999	Mowing/grazing	Beginning of August	After mowing	

References

Hallgren G., Sandsborg J. On the variation in groundwater level in the Uppsalaåsen esker in the years 1938–1967. Grundförbättring 1968;21: 85–131. Swedish.

Hebbe P. [Some notes from Ultuna's oldest history]. Stockholm: Kongl. Landtbruks-akademien; 1936. Swedish.

Länsstyrelsen i Uppsala län. [The Administrative Board of Uppsala County resolution in connection with … "Kungsängen nature reserve" in the parish of Danmark; given at Uppsala Castle in the National Chancellery on March 30, 1951]; 1951. Swedish.

Länsstyrelsen i Uppsala län. [Establishment of management plan for the nature reserve Uppsala Kungsäng, Uppsala kommun]; 1999. Swedish.

Mälarens vattenvårdsförbund. The regulation of Lake Mälaren. Available at http://www.malaren.org/malaren/nyttjande-och-paverkan/fysisk-paverkan/regleringen-av-malaren [Accessed 14 March 2018]. Mälarens vattenvårdsförbund; 2014. Swedish.

Rudbeck O. Atlantica. Facsimile edition of the Atlantica published by Axel Nelson 1937–1950. Uppsala: Almqvist & Wiksell; 1950 [1679].

Sandberg G. [Regional studies of Kungsängen's vegetation, as well as views on the nature of our meadows and protection issues]. In: Sernander R. Uppsala Kungsäng. 1948. p. 145–210. Swedish.

Sernander R. Uppsala Kungsäng. Published by Gustaf Sandberg. Uppsala: Almqvist & Wiksell; 1948. Swedish.

SMHI. Facts about Lake Mälaren. Available at https://www.smhi.se/kunskapsbanken/hydrologi/fakta-om-malaren-1.5089 [Accessed 18 March 2018]. SMHI; 2012. Swedish.

Stockholms hamnar. Lake Mälaren's regulation. Available at https://www.stockholmshamnar.se/historia/organisation/malarens-reglering [Accessed 28 April 2021]. Stockholms hamnar; 2021. Swedish.

Yckenberg P, Lindberg O. Geometric map of Ultuna Kongs Ladugård. Lantmäteristyrelsen; 1732–1735. Swedish.

Zhang L. Vegetation ecology and population biology of Fritillaria meleagris L. at the Kungsängen nature reserve, Eastern Sweden. Acta Phytogeogr Suec 1983;73: 1–96.

Zhang L, Hytteborn H. Effect of ground water regime on development and distribution of Fritillaria meleagris. Holarct Ecol 1985;8: 237–44. https://doi.org/10.1111/j.1600-0587.1985.tb01174.x
