# Supplementary material for: Spatial heterogeneity ensures long-term stability in vegetation and Fritillaria meleagris flowering in Uppsala Kungsäng, a semi-natural meadow
Source: PLoS One. 2023 Mar 8;18(3):e0282116. doi: 10.1371/journal.pone.0282116 (PMC10045606; doi:10.1371/journal.pone.0282116)
Supplement: S3 Appendix — (PDF) [file pone.0282116.s003.pdf]

Appendix S3. Anova of indicator values in Profile 1, 1938-2016.

Mixed repeated-measures analyses of abundance-weighted indicator values for Moisture, Light, Nitrogen, Grazing/mowing, Soil disturbance and Soil reaction in Profile 1. ”Group” compares plots in the Wet and Mesic groups.

Moisture

Source	Numerator df	Denominator df	F	P
Group	1	76.677	219.729	0.000
Year	3	37.113	26.891	0.000
Group × Year	3	37.113	43.519	0.000

Light

Source	Numerator df	Denominator df	F	P
Group	1	2.103	44.963	0.019
Year	3	40.272	4.043	0.013
Group × Year	3	40.272	1.874	0.149

Nitrogen

Source	Numerator df	Denominator df	F	P
Group	1	76.579	5.594	0.021
Year	3	41.744	17.083	0.000
Group × Year	3	41.744	1.276	0.295

Grazing/mowing

Source	Numerator df	Denominator df	F	P
Group	1	83.383	186.044	0.000
Year	3	50.129	2.717	0.054
Group × Year	3	50.129	11.027	0.000

Soil disturbance

Source	Numerator df	Denominator df	F	P
Group	1	77.570	0.604	0.439
Year	3	47.488	13.965	0.000
Group × Year	3	47.488	20.184	0.000

Soil reaction

Source	Numerator df	Denominator df	F	P
Group	1	12.189	2.329	0.153
Year	3	36.767	4.818	0.006
Group × Year	3	36.767	2.766	0.056
