# Supplementary material for: Spatial heterogeneity ensures long-term stability in vegetation and Fritillaria meleagris flowering in Uppsala Kungsäng, a semi-natural meadow
Source: PLoS One. 2023 Mar 8;18(3):e0282116. doi: 10.1371/journal.pone.0282116 (PMC10045606; doi:10.1371/journal.pone.0282116)
Supplement: S4 Appendix — (PDF) [file pone.0282116.s004.pdf]

Appendix S4. Anova of indicator values in Profiles 1–4, 1982-2016.

Mixed repeated-measures analyses of abundance-weighted indicator values for Moisture, Light, Nitrogen, Grazing/mowing, Soil disturbance and Soil reaction (pH) in Profiles 1–4. "Cluster" compares plots from the four clusters defined in the NMDS ordination.

Moisture

Source	Numerator df	Denominator df	F	P
Cluster	3	203.557	437.001	<.001
Year	2	132.191	3.968	.021
Cluster × Year	6	132.191	1.094	.369

Light

Source	Numerator df	Denominator df	F	P
Cluster	3	194.395	23.100	<.001
Year	2	126.368	3.154	.046
Cluster × Year	6	126.368	2.582	.022

Nitrogen

Source	Numerator df	Denominator df	F	P
Cluster	3	206.682	17.159	<.001
Year	2	148.859	6.929	.001
Cluster × Year	6	148.859	3.574	.002

Grazing/mowing

Source	Numerator df	Denominator df	F	P
Cluster	3	170.052	283.660	<.001
Year	2	117.724	2.229	.112
Cluster × Year	6	117.724	1.395	.222

Soil disturbance

Source	Numerator df	Denominator df	F	P
Cluster	3	210.978	141.366	<.001
Year	2	137.424	3.074	.049
Cluster × Year	6	137.424	.806	.567

Soil reaction

Source	Numerator df	Denominator df	F	P
Cluster	3	191.357	11.646	<.001
Year	2	144.888	.837	.435
Cluster × Year	6	144.888	1.101	.365
