# Supplementary material for: Spatial heterogeneity ensures long-term stability in vegetation and Fritillaria meleagris flowering in Uppsala Kungsäng, a semi-natural meadow
Source: PLoS One. 2023 Mar 8;18(3):e0282116. doi: 10.1371/journal.pone.0282116 (PMC10045606; doi:10.1371/journal.pone.0282116)
Supplement: S5 Appendix — (PDF) [file pone.0282116.s005.pdf]

Appendix S5. Number of flowering *Fritillaria meleagris* at Uppsala Kungsäng.

Number of flowering *Fritillaria meleagris* in six 10 m × 10 m plots (Fig. 1; total area 600 m^2), in 1938, 1981–1988 and 2016–2021. The proportion of white and pink flowers was not recorded in 2016. Data for 1938 from Sernander (1948).

Elevation (m a.s.l.) Year	Plot 1 1.83	Plot 2 1.84	Plot 3 1.84	Plot 4 1.86	Plot 5 1.57	Plot 6 1.31	Total	White (%)	Pink (%)
1938	11	19	149	452	1932	255	2818	3.5	1.8
1981	16	66	98	2145	48	0	2373	3.0	1.1
1982	39	243	274	1132	7	0	1695	3.5	2.8
1983	61	291	239	1862	152	0	2605	4.2	1.8
1984	17	344	334	2138	260	6	3099	3.3	2.3
1985	92	726	813	2530	281	10	4452	3.5	2.1
1986	31	361	325	1699	362	0	2778	2.4	1.3
1987	59	314	442	1013	505	8	2341	4.5	2.1
1988	74	317	528	678	341	22	1960	3.4	1.1
2016	6	36	76	701	1040	54	1913	–	–
2017	13	56	96	760	730	67	1722	4.5	0.8
2018	39	204	256	1403	1244	160	3306	4.0	2.9
2019	22	64	104	605	895	155	1845	4.0	2.4
2020	23	162	249	836	1363	189	2822	3.5	1.2
2021	47	311	497	1111	2218	335	4519	2.6	1.0

Reference

Sernander R. Uppsala Kungsäng. Published by Gustaf Sandberg. Uppsala: Almqvist & Wiksell; 1948. Swedish.
