# Supplementary material for: Spatial heterogeneity ensures long-term stability in vegetation and Fritillaria meleagris flowering in Uppsala Kungsäng, a semi-natural meadow
Source: PLoS One. 2023 Mar 8;18(3):e0282116. doi: 10.1371/journal.pone.0282116 (PMC10045606; doi:10.1371/journal.pone.0282116)
Supplement: S6 Appendix — (PDF) [file pone.0282116.s006.pdf]

Appendix S6. Matrix analyses of *Fritillaria meleagris* 1981–1983.

Transition matrices

Transition matrices for the years 1981–1982 and 1982–1983 for a population of *Fritillaria meleagris* followed at Uppsala Kungsäng for three consecutive years (1981–1983). The stages are small vegetative plants, medium sized vegetative plants, large vegetative plants, reproductive plants, and plants that entered prolonged dormancy (Dormant). Number of individuals (including dead plants) in each stage was 580, 258, 494, 405 and 798 for the transition 1981–1982, and 652, 228, 692, 223 and 881 for the transition 1982–1983.

1981-1982

	Small veg.	Medium veg.	Large veg.	Repr.	Dormant
Small veg.	0.5224	0.0775	0.0182	0.0049	0.4837
Medium veg.	0.1052	0.3256	0.0385	0.0494	0.0865
Large veg.	0.0241	0.4186	0.5202	0.6000	0.1679
Repr.	0.0017	0.0155	0.2429	0.2247	0.0213
Dormant	0.1431	0.0891	0.1154	0.0815	0

1982-1983

	Small veg.	Medium veg.	Large veg.	Repr.	Dormant
Small veg.	0.5706	0.0877	0.0058	0	0.5743
Medium veg.	0.1150	0.3728	0.0462	0.0448	0.0874
Large veg.	0.0291	0.3904	0.5072	0.4170	0.1646
Repr.	0	0.0395	0.3772	0.4798	0.0182
Dormant	0.1181	0.0614	0.0405	0.0404	0

Sensitivity matrices

Sensitivity matrices for the years 1981–1982 and 1982–1983 for a population of *Fritillaria meleagris* followed at Uppsala Kungsäng for three consecutive years (1981–1983). In bold are those transitions where changes in the transition matrices would have the largest impact on long-term population growth rates.

1981-1982

	Small veg.	Medium veg.	Large veg.	Repr.	Dormant
Small veg.	0.1244	0.0631	0.2865	0.1083	0.0732
Medium veg.	0.2034	0.1032	**0.4687**	0.1772	0.1197
Large veg.	0.2158	0.1095	**0.4972**	0.1880	0.1270
Repr.	0.2261	0.1147	**0.5208**	0.1969	0.1330
Dormant	0.1331	0.0675	0.3066	0.1159	0.0783

1982-1983

	Small veg.	Medium veg.	Large veg.	Repr.	Dormant
Small veg.	0.0568	0.0489	0.2351	0.1916	0.0282
Medium veg.	0.0964	0.0830	**0.3990**	**0.3253**	0.0479
Large veg.	0.1098	0.0945	**0.4544**	**0.3705**	0.0546
Repr.	0.1109	0.0954	**0.4588**	**0.3740**	0.0551
Dormant	0.0640	0.0551	0.2649	0.2159	0.0318
