# Supplementary material for: Spatial heterogeneity ensures long-term stability in vegetation and Fritillaria meleagris flowering in Uppsala Kungsäng, a semi-natural meadow
Source: PLoS One. 2023 Mar 8;18(3):e0282116. doi: 10.1371/journal.pone.0282116 (PMC10045606; doi:10.1371/journal.pone.0282116)
Supplement: S7 Appendix — (PDF) [file pone.0282116.s007.pdf]

Appendix S7. Species with a significant change in cover between the first and last survey.

Species with a significant change in cover between the first and last survey (1940–2016 in Profile 1, $n = 28$; 1982–2016 in Profiles 1–4, $n = 76$). Numbers are mean cover, with plot frequency in parenthesis. For each category the species with < 1% in mean cover are listed at the end. Asterisks indicate species with a significant ($p < 0.05$) change in frequency in the same direction as in cover. Two species that increased in cover but decreased in frequency are indicated by *-.

Species	Cover 1940 (%)	Cover 1982 (%)	Cover 1995 (%)	Cover 2016 (%)
Profile 1 1940–2016, decreasing				
Argentina anserina*	4.1 (15)	0.7 (6)	0.5 (5)	0.1 (1)
Deschampsia cespitosa*	21.8 (26)	11.4 (20)	6.5 (19)	2.6 (14)
Elytrigia repens*	9.2 (21)	1.7 (6)	0.2 (2)	1.2 (10)
Poa pratensis*	9.3 (23)	4.0 (18)	10.3 (21)	3.1 (9)
Ranunculus auricomus*	2.1 (21)	1.7 (18)	1.9 (21)	1.0 (10)
Ranunculus repens*	2.8 (19)	0.7 (5)	0.5 (6)	0.6 (6)
Decreasing (<1% cover)				
Agrostis canina	0.5 (5)	- (0)	- (0)	- (0)
Lysimachia nummularia*	0.7 (10)	0.5 (5)	- (0)	0.1 (1)
Profile 1 1940–2016, increasing				
Carex acuta*	6.4 (5)	16.3 (11)	15.9 (10)	16.3 (12)
Carex cespitosa*	- (0)	3.1 (6)	2.7 (5)	9.3 (8)
Filipendula ulmaria	0.5 (7)	1.6 (13)	1.6 (13)	3.1 (9)
Persicaria amphibia*	- (0)	1.6 (17)	1.1 (12)	0.6 (6)
Phleum pratense	0.9 (14)	1.3 (12)	4.5 (19)	5.1 (16)
Poa trivialis	0.9 (11)	1.1 (12)	1.5 (16)	1.7 (17)
Stellaria graminea*	0.5 (7)	1.0 (11)	1.4 (15)	1.6 (16)
Trifolium pratense*	- (0)	1.0 (11)	1.3 (14)	3.0 (12)
Trifolium repens*	0.3 (4)	1.6 (17)	1.6 (17)	6.1 (15)
Vicia cracca*	1.2 (16)	1.8 (19)	1.7 (19)	3.7 (16)
Increasing (<1% cover)				
Anthriscus sylvestris	- (0)	0.6 (6)	0.8 (9)	0.2 (2)
Cirsium arvense*	- (0)	- (0)	- (0)	0.8 (8)
Lemna minor	- (0)	0.7 (7)	- (0)	0.4 (4)
Vicia sepium*	- (0)	- (0)	- (0)	0.3 (3)
Profiles 1–4 1982–2016, decreasing				
Avenula pubescens*	-	2.3 (34)	1.6 (32)	0.9 (21)
Cardamine pratensis*	-	1.0 (28)	0.5 (16)	0.2 (4)
Deschampsia cespitosa*	-	8.3 (58)	6.4 (49)	2.4 (33)
Galium uliginosum*	-	1.2 (35)	0.8 (25)	0.9 (23)
Persicaria amphibia*	-	1.7 (49)	1.2 (35)	0.7 (19)
Ranunculus auricomus*	-	1.8 (54)	2.1 (63)	1.2 (31)
Decreasing (<1% cover)				
Agrostis stolonifera*	-	0.7 (19)	- (0)	0.1 (2)
Alchemilla sp.*	-	0.5 (16)	0.4 (13)	0.3 (7)
Argentina anserina*	-	0.6 (16)	0.4 (12)	0.2 (4)
Capsella bursa-pastoris*	-	0.3 (8)	0.1 (3)	<0.1 (1)
Carex sp.*	-	0.4 (13)	0.5 (14)	- (0)
Carum carvi*	-	0.2 (7)	<0.1 (1)	- (0)
Cirsium vulgare*	-	0.3 (9)	0.3 (10)	<0.1 (1)
Eleocharis palustris	-	0.1 (4)	- (0)	- (0)

Galium boreale*	-	0.8 (24)	0.9 (26)	0.5 (10)
Heracleum sphondylium	-	0.2 (5)	0.2 (7)	- (0)
Lemna minor*	-	0.3 (10)	- (0)	0.2 (4)
Lemna trisulca*	-	0.3 (10)	- (0)	0.1 (3)
Lotus corniculatus	-	0.1 (3)	- (0)	- (0)
Lysimachia nummularia*	-	0.7 (22)	0.1 (2)	0.2 (5)
Lysimachia thyrsiflora*	-	0.4 (11)	0.4 (12)	0.1 (2)
Matricaria discoidea*	-	0.2 (7)	<0.1 (1)	- (0)
Oenanthe aquatica	-	0.1 (4)	- (0)	- (0)
Phalaris arundinacea*	-	0.4 (10)	0.3 (7)	0.1 (2)
Plantago major*	-	0.3 (10)	<0.1 (1)	<0.1 (1)
Poa annua*	-	0.2 (6)	0.1 (3)	- (0)
Polygonum aviculare*	-	0.2 (6)	<0.1 (1)	- (0)
Ranunculus polyanthemos	-	0.1 (4)	0.1 (3)	- (0)
Ranunculus sceleratus	-	0.1 (4)	0.1 (4)	- (0)
Saxifraga sp.*	-	0.2 (6)	0.1 (3)	- (0)
Stellaria palustris*	-	0.5 (14)	0.5 (16)	0.2 (4)
Tripleurospermum inodorum*	-	0.3 (8)	<0.1 (1)	- (0)
Veronica sp.*	-	0.3 (10)	<0.1 (1)	- (0)
Profiles 1–4 1982–2016, increasing				
Carex cespitosa	-	3.2 (19)	1.5 (14)	8.5 (21)
Cirsium arvensis*	-	- (0)	- (0)	1.0 (21)
Elytrigia repens*	-	1.3 (15)	1.3 (19)	3.8 (39)
Filipendula ulmaria*-	-	2.0 (45)	1.9 (39)	6.0 (32)
Lathyrus pratensis	-	1.6 (47)	1.9 (57)	2.7 (48)
Schedonorus pratensis	-	1.0 (29)	1.7 (40)	3.1 (34)
Stellaria graminea*	-	0.8 (24)	1.2 (35)	1.5 (37)
Trifolium pratense	-	1.2 (32)	1.4 (35)	2.7 (28)
Trifolium repens*-	-	2.1 (44)	1.6 (43)	5.6 (32)
Vicia cracca	-	2.1 (55)	2.2 (58)	3.8 (49)
Increasing (<1% cover)				
Cirsium sp.	-	- (0)	- (0)	0.1 (3)
Galium aparine	-	- (0)	- (0)	0.2 (4)
Galium mollugo	-	- (0)	- (0)	0.1 (3)
Vicia sepium	-	- (0)	- (0)	0.1 (3)
