# Supplementary material for: Spatial heterogeneity ensures long-term stability in vegetation and Fritillaria meleagris flowering in Uppsala Kungsäng, a semi-natural meadow
Source: PLoS One. 2023 Mar 8;18(3):e0282116. doi: 10.1371/journal.pone.0282116 (PMC10045606; doi:10.1371/journal.pone.0282116)
Supplement: S8 Appendix — (PDF) [file pone.0282116.s008.pdf]

Appendix S8. Generalised linear mixed models of the effects of climate and elevation on the number of *Fritillaria meleagris* flowers.

Effects of climate (temperature and precipitation) and elevation on the number of *Fritillaria meleagris* flowers tested by General Linear Mixed Models.

	Temperature March-April		Temperature Previous June		Temperature Previous September		Precipitation March-April		Precipitation Previous June		Precipitation Previous September	
Parameter	Coeff ± SE	*P*	Coeff ± SE	*P*	Coeff ± SE	*P*	Coeff ± SE	*P*	Coeff ± SE	*P*	Coeff ± SE	*P*
Intercept	5.31 ± 0.498	< 0.001	5.38 ± 0.491	< 0.001	5.42 ± 0.488	< 0.001	5.48 ± 0.485	< 0.001	5.39 ± 0.491	< 0.001	5.48 ± 0.484	< 0.001
Climate	0.12 ± 0.006	< 0.001	0.20 ± 0.005	< 0.001	0.11 ± 0.006	< 0.001	-0.01 ± 0.005	0.021	-0.04 ± 0.006	< 0.001	0.07 ± 0.005	< 0.001
Elevation	0.50 ± 0.501	0.317	0.44 ± 0.494	0.373	0.36 ± 0.491	0.46	0.28 ± 0.487	0.571	0.36 ± 0.493	0.461	0.26 ± 0.487	0.600
C × E	-0.64 ± 0.008	< 0.001	-0.44 ± 0.007	< 0.001	-0.41 ± 0.008	< 0.001	0.19 ± 0.007	< 0.001	0.51 ± 0.008	< 0.001	0.17 ± 0.007	< 0.001
Marginal R^2	0.31		0.23		0.18		0.07		0.21		0.07	
