# Supplementary material for: Spatial heterogeneity ensures long-term stability in vegetation and Fritillaria meleagris flowering in Uppsala Kungsäng, a semi-natural meadow
Source: PLoS One. 2023 Mar 8;18(3):e0282116. doi: 10.1371/journal.pone.0282116 (PMC10045606; doi:10.1371/journal.pone.0282116)
Supplement: Attachment — Submitted filename: Response to Reviewers R2.docx [file pone.0282116.s010.docx]

Dear Editor,

We now submit a second revised version of the manuscript PONE-D-22-16087, entitled 'Spatial heterogeneity ensures long-term stability in vegetation and *Fritillaria meleagris* flowering in Uppsala Kungsäng, a semi-natural meadow'. Below we describe how we have dealt with each of the comments from the Editor and the two reviewers.

For the author team,

Håkan Rydin

***********************************************************************

# Comments from the Editor

Thank you for submitting your manuscript to PLOS ONE. After careful consideration, we feel that it has merit but does not fully meet PLOS ONE’s publication criteria as it currently stands. Therefore, we invite you to submit a revised version of the manuscript that addresses the points raised during the review process.

Both reviewers note the revised manuscript is significantly improved over the original submission and acknowledge your efforts to address all their concerns. Most remaining issues deal with problems of clarity and flow/structure, although I identify a couple other needed areas for improvement in the list below.

- Abstract. Structure, content, and clarity are lacking. There are many guides and publications available that one can consult to get a good sense of the format of a strong Abstract, e.g. <https://guides.lib.uci.edu/c.php?g=334338&p=2249902>.

*Response: Thanks for the hint!* *The Abstract is re-written with Editor’s and Reviewers’ comments in mind.*

Also, some text is strangely worded, which adds to the lack of clarity, e.g. Lines 18-21. What exactly do you mean by active periods? Do you mean when the species is flowering? Regardless, doesn't temperature and precipitation dictate active periods/flowering versus the other way around? As written, the content of the sentence seems backwards. In the next sentence, what do you mean by 'Thereby'? I assume what you're trying to say is that because of this annual and spatial variation in flowering due to temp, precip, and wet/dry areas of the meadow, there was significant year to year variation in flowering. But maybe not. Be explicit rather than using a short hand word like 'thereby'.

*Response: We have replaced ‘active periods’ with defining the phenological phases of the plant, so it should now be clear that weather in these periods (June previous year, September previous year, and March to April) affects flowering (in May). The sentence with ‘thereby’ is re-phrased.*

Line 23-26. Add a comma after 'composition' and 'the' before 'long-term'. That will help clarify that richness and composition refer to the meadow vegetation and not the Fritillaria population (e.g. Reviewer 1 comment that populations don't have richness). You might also consider rewording to make it even more explicit that the first part of that sentence refers to meadow vegetation. Also, seems 'long-term in...' should be 'long-term of...' What do you mean by 'upheld'?

*Response: Changed to “Species richness and species composition of the meadow vegetation, and the long-term stability of the F. meleagris population are maintained by…”.*

- Materials & Methods. Clarity/flow of this section would be improved by additional structure in the form of subsections. As Reviewer 2 noted, statistical approaches are commonly included in a separate subsection.

*Response: We have re-arranged Material and methods as suggested by Reviewer 2*

- Species names. On first use and when used at the beginning of a sentence fully spell out both Genus and species. On subsequent use, abbreviate Genus, e.g. Carex acuta when first stated, C. acuta elsewhere unless it starts a sentence. Please check that your use of all species names follows this rule.

*Response: Fixed.*

- White morph. For clarity be explicit that 'flower-picking townsfolk' are agents of natural selection on flower color because of their preference for white flowers (e.g. Reviewer 1 comment).

*Response: It was actually Reviewer 2 who commented on this. We clarify that the flower-picking “may have decreased its relative frequency in the population by reducing recruitment from seeds”.*

- Line 252-253. Why is font size different here?

*Response: Fixed.*

- References. Italicize species names throughout (they currently aren't). Line 831. Delete extra space before 'term'. Ensure that spacing and format used is consistent throughout, e.g. page numbering isn't, Line 838 vs 840.

*Response: Fixed.*

# Reviewers' comments:

1. If the authors have adequately addressed your comments raised in a previous round of review and you feel that this manuscript is now acceptable for publication, you may indicate that here to bypass the “Comments to the Author” section, enter your conflict of interest statement in the “Confidential to Editor” section, and submit your "Accept" recommendation.

Reviewer #1: All comments have been addressed

Reviewer #2: All comments have been addressed

2. Is the manuscript technically sound, and do the data support the conclusions?

The manuscript must describe a technically sound piece of scientific research with data that supports the conclusions. Experiments must have been conducted rigorously, with appropriate controls, replication, and sample sizes. The conclusions must be drawn appropriately based on the data presented.

Reviewer #1: Yes

Reviewer #2: Yes

3. Has the statistical analysis been performed appropriately and rigorously?

Reviewer #1: Yes

Reviewer #2: I Don't Know

4. Have the authors made all data underlying the findings in their manuscript fully available?

The [PLOS Data policy](http://www.plosone.org/static/policies.action#sharing) requires authors to make all data underlying the findings described in their manuscript fully available without restriction, with rare exception (please refer to the Data Availability Statement in the manuscript PDF file). The data should be provided as part of the manuscript or its supporting information, or deposited to a public repository. For example, in addition to summary statistics, the data points behind means, medians and variance measures should be available. If there are restrictions on publicly sharing data—e.g. participant privacy or use of data from a third party—those must be specified.

Reviewer #1: Yes

Reviewer #2: Yes

5. Is the manuscript presented in an intelligible fashion and written in standard English?

PLOS ONE does not copyedit accepted manuscripts, so the language in submitted articles must be clear, correct, and unambiguous. Any typographical or grammatical errors should be corrected at revision, so please note any specific errors here.

Reviewer #1: Yes

Reviewer #2: Yes

# Reviewer #1:

This manuscript is much improved from the previous draft and suggested edits have been addressed. The addition of elevation from lidar is very valuable and the discussion is more interesting and broader. The manuscript is still quite long and detailed but rejigging the methods would help improve the flow. I have some minor comments below, which are largely for clarity and improving understanding for the readers.

Abstract

I think the abstract is lacking a so what, why part. Your first sentence is a general one about management but then the next is about analysing the vegetation. There needs to be a sentence in the middle bringing together what your study is addressing.

*Response: The Abstract is re-written with Editor’s and Reviewers’ comments in mind. We have now toned down the management aspect in the first sentence to more directly focus on the research question: that the vegetation may have been affected by environmental change and altered management.*

Line 14: This wording is slightly odd, perhaps something like “Management is crucial for maintaining the richness and diversity of semi-natural grasslands”.

*Response: Deleted as a result of the previous comment.*

Line 33: I don’t think you need to say “overall”

*Response: Deleted.*

Introduction

Line 45: I wouldn’t mention “such as the one studied in this paper”. Its nice to keep the first paragraph in the introduction general.

*Response: Deleted.*

Line 46: You mention several elements but only state two – mowing and grazing.

*Response: Simplified as “traditional management usually involves mowing in mid- to late summer and aftermath grazing”*

Line 52: Do you have a reference to support this?

*Response: We have added a reference.*

Line 55: What is a general impoverishment of flora? Loss of species diversity? Loss of species richness?

*Response: Changed to “loss of species diversity and a more trivial flora”.*

Line 63: Reference needed here. You could also add here that meadows and semi-natural grasslands are important for the delivery of ecosystems e.g. pollination, carbon storage etc. See Bengtsson et al (2019) Ecosphere.

*Response: Thanks, we incorporated ecosystem services, supported by this reference.*

Figure 1 is much improved. However, I would show the elevation scale with a legend on the map rather than written in the legend, as its quite harder for a reader to understand what the elevation of yellow areas are for example.

*Response: Elevation scale added.*

Line 101-103: These sentences seem a bit random. Perhaps is worth moving these until after you’ve described the history of the site.

*Response: Yes, we did so.*

Method

I think it would be clearer to rejig the methods. Personally, I think it would be clearer to have “1. Vegetation Survey” – here include the general vegetation survey and F.meleagris survey too. Then have “2. Statistical analysis” – this could be split in 2.1 Vegetation change and 2.2. Variation in flowering.

*Response: Re-arranged as suggested.*

Line 313: This would be more appropriately placed in the statistical analysis, when you mention about using elevation.

*Response: Done*

Line 346: What source did you use for the six indicators? Reference needed.

*Response: Reference to Tyler’s paper added.*

Line 401: Good to provide a reference for why you centred and scale predictor variables e.g. Schielzeth, H. 2010. Simple means to improve the interpretability of regression coefficients. – Methods Ecol. Evol. 1: 103–113.

*Response: Nice reference, since the answer is in its title! We added it.*

394: So for climate were both temperature and precipitation used? Did you check if these were correlated?

*Response: We have now added that the two predictors were uncorrelated (with p-values)*

Line 406: Perhaps make this a bit clearer by stating we eliminated these three years and thus the analysis was based on the remaining 12 years of data.

*Response: Changed as suggested.*

Results

Table 1: I’m assuming this is the average species richness and diversity? It would be good to say that and provide standard error or deviation with this.

*Response: No, since this table is meant to show overall changes it is total species richness in these plots. So we changed the caption to “Total species richness…”.*

Fig 2: You’ve labelled the plots now so you can remove the “w” and “m” part from your written figure legend. It seems a bit random to just “include some other species mentioned in the text”, I would be tempted to just leave it as the 6 most abundant (same for Figure 5a).

*Response: labels “w” and “m” are removed. The choice of species may seem random, but they are not arbitrarily selected. In the text we mention, for example, ruderal species (and were asked by reviewer to add some more), and it is crucial for the interpretation to show these in the ordination. To avoid the appearance of randomness, we changed to “some other indicative species (such as ruderals)”. Same in Fig. 5.*

Fig 9: Why were these elevation values selected?

*Response: Not selected; the numbers (1.83, 1.84 etc.) are the actual elevation values for each plot. We think this is clear from the legend.*

Fig 10: How were high and low elevation split?

*Response: We did not 'split' the dataset into high and low elevation plots. Rather, elevation was treated as a continuous variable in our models. To better visualize the interactive effects of climate and elevation, we show predictions of our model across the climate axis for the highest and lowest elevations included in the data.*

Discussion

This section is much improved.

Line 714: Vegetation change or change in vegetation would be more consistent with your results subheadings.

*Response: Changed to “Changes in vegetation” as in Methods and Results.*

Line 750-762: It might be nitrogen deposition and grazing/mowing are interacting. Its hard to disentangle the two – see Wilson E, Wells T, Sparks T (1995) Are calcareous grasslands in the UK under threat from nitrogen deposition? -an experimental determination of a critical load. J Ecol 83:823–832

*Response: True, so we added “Nitrogen effects are often confounded with effects of management and can be difficult to disentangle” with the suggested reference (and one more).*

Line 1062: And I guess the importance of ensuring that some management takes place even if its not the traditional management that used to take place?

Response: *The last sentence is changed to “…highlighting the importance of spatial heterogeneity and appropriate management…”.*

# Reviewer #2:

Good work on revision of the paper! I still feel it contains at least two papers in one. Still too much emphasis on management, in places.

*Response: We toned down management in the opening of the abstract. See also response to comment on the Introduction below.*

Still, no biological justification to include flower color analysis in the paper. Ratio of white/pink flowers is population dependent, genetically driven, nothing to do with picking up white flowers by visitors of the meadow.

*Response: We agree with the Editor’s argument that selective ‘flower-picking townsfolk' are agents of natural selection on flower color because of their preference for white flowers. In the text we clarify that this selective picking of white flowers “may have decreased its relative frequency in the population by reducing recruitment from seeds”.*

An opening sentence of the Abstract states importance of management, while the title of the paper: role of spatial heterogeneity. It would be logical to start Abstract with the statement relevant to the topic.

*Response: The Abstract is re-written with Editor’s and Reviewers’ comments in mind. We have now toned down the management aspect in the first sentence to more directly focus on the research question: that their vegetation may have been affected by environmental change and altered management.*

"Species richness, overall species composition and long-term stability in the Fritillaria meleagris population" ----- A population does not have species richness or species composition…

*Response: Changed to “Species richness and species composition of the meadow vegetation, and the long-term stability of the F. meleagris population…”.*

Introduction has been largely improved, looks much better. Only, once again, management there has been stated very strongly from the very beginning, while hydrology and spatial heterogeneity are not presented as the main drivers of the vegetation in the meadows. I still would suggest to focus more on the topic. E.g., floodplain meadows developed substantial heterogeneity within their landscape… ancient channels, flood water ponds, natural elevation gradient, etc. They have been used as prime habitats for hay making in summer and grazing in… , sustainable biomass production because of nutrients arriving with the floods,… Lack of hay cut/grazing – deposit of dead biomass (litter) preventing seedling recruitments…

*Response: We have made an attempt to restructure the Introduction along these lines.*

Line 597-598 and below

Again, a very interesting discussion about weather effect on flowering of Fritillaria meleagris. The species seasonal development is very well studied quite long time ago. See

<http://www.fritillariaicones.com> › info › Baranova

If to discuss weather effects on population dynamics, it would be most useful to apply existing knowledge about different life stages to weather at different times of year to follow actual relationship between two. I think, this is a good data and idea for paper on its own.

*Response: We have added the suggested reference in the Introduction. We agree that another paper is possible, and we are considering to model flowering in response to future climate scenarios.*
