# Supplementary material for: Spatial heterogeneity ensures long-term stability in vegetation and Fritillaria meleagris flowering in Uppsala Kungsäng, a semi-natural meadow
Source: PLoS One. 2023 Mar 8;18(3):e0282116. doi: 10.1371/journal.pone.0282116 (PMC10045606; doi:10.1371/journal.pone.0282116)
Supplement: Attachment — Submitted filename: Response to Reviewers R3.docx [file pone.0282116.s011.docx]

Dear Editor,

We now submit a third revised version of the manuscript PONE-D-22-16087, entitled 'Spatial heterogeneity ensures long-term stability in vegetation and *Fritillaria meleagris* flowering in Uppsala Kungsäng, a semi-natural meadow'. Below we describe how we have dealt with each of the comments from the Editor.

For the author team,

Håkan Rydin

***********************************************************************

# Comments from the Editor

Thank you for submitting your manuscript to PLOS ONE.  Although revisions have greatly improved the manuscript, there are a few very minor issues that remain to be addressed before it can be moved forward.  Please revise accordingly and resubmit.

Captions for Figure 2 & 5:  "...some other indicative species..."  Like Reviewer 1, I stumble over the phrasing and meaning here.  For one, please change "indicative' to 'indicator'.  Indicator species is a commonly used phrase but indicative species is not.  Second,  the use of "...some other..." doesn't fully clarify the problem that Reviewer 1 identified.  I tried toying around with some modifications, but wasn't completely happy with any.  Maybe: "...six most abundant species in each envelope and other representative indicator species that were mentioned in the text (such as ruderals)".  See if you can further tweak text for Caption Fig 2 and 5 to increase clarity.  Regardless, the same text should be added to both caption, e.g. Fig 5 caption doesn't currently include the clarifying bit of text, "such as ruderals".

*Response: We appreciate your efforts in finding a way to clarify what we mean. We follow your suggestion and have changed to “…and other representative species that are mentioned in the text (such as ruderals).” We avoid using “indicator” as this word is associated with Ellenberg indicator values in the manuscript; these species are in the text.*

Line 305:  R.acris.  Insert a space after the period, delete the space after 's'.

*Response: Corrected.*

Line 316:  R.auricomus.  Insert a space after the period.

*Response: Corrected.*

Line 647:  Please change 'upheld' to 'maintained'.

*Response: Changed as suggested.*
